# The tibialis anterior tendon revisited: a unified classification framework across development, evolution, and clinical application

**DOI:** 10.3389/fcell.2025.1678982

**Published:** 2025-10-08

**Authors:** Łukasz Olewnik, Ingrid C. Landfald, Magdalena Ciechanowska, Bento João Abreu, Judney Cley Cavalcante

**Affiliations:** ^1^ Department of Clinical Anatomy, Mazovian Academy in Plock, Plock, Poland; ^2^ VARIANTIS Research Laboratory, Mazovian Academy in Plock, Plock, Poland; ^3^ Group of Study and Research in Human Anatomy (NEPAH), Department of Morphology, Federal University of Rio Grande do Norte, Rio Grande, Brazil

**Keywords:** tibialis anterior tendon, anatomical variation, developmental classification, musculoskeletal ultrasound, comparative anatomy, tendon transfer, lower limb

## Abstract

The tibialis anterior tendon (TAT), the terminal extension of the tibialis anterior muscle (TAM), plays a key role in dorsiflexion and inversion of the foot. Although the TAM exhibits morphological constancy, its tendon demonstrates substantial variability in distal insertion patterns, with direct implications for radiological interpretation, surgical approaches, and anatomical education. This review synthesizes evidence from developmental anatomy, cadaveric dissection, and high-resolution imaging to propose a unified six-type classification of the TAT. The framework integrates fetal, adult, and ultrasonographic findings and highlights the significance of Types V and VI as the most surgically accessible and structurally consistent variants. Bifid and trifid insertion patterns (Types I–IV) may contribute to mediolateral foot control and demonstrate functional adaptation, particularly in the context of human bipedal locomotion. Comparative anatomical analysis across vertebrates reveals an evolutionary trajectory from simple dorsal muscle structures in amphibians and reptiles to specialized bifid insertions in primates and humans. Type VI may represent a recently derived morphology with possible functional redundancy. From a diagnostic standpoint, knowledge of TAT variants is essential to prevent misinterpretation of anatomical bifurcations as tendinopathy or partial tears in ultrasound and MRI assessments. Clinically, the classification aids in tendon transfer planning, especially for foot drop correction and reconstructive procedures. We recommend this unified classification as a reference standard for anatomical teaching, clinical diagnostics, and preoperative decision-making. Future research should include three-dimensional modeling of insertion geometry, biomechanical simulations of tendon function across types, and longitudinal studies tracking the ontogeny of TAT morphology.

## 1 Introduction

The tibialis anterior muscle (TAM) is the principal dorsiflexor and inverter of the foot, playing a crucial role in maintaining postural balance, particularly during the stance and swing phases of gait (Day et al., 2013; Maharaj et al., 2019). Its muscle belly demonstrates a consistent anatomical configuration across individuals, originating from the lateral condyle and proximal two-thirds of the lateral surface of the tibia, as well as the interosseous membrane and deep fascia of the leg (Moore et al., 2017; Spence and Forro, 2020).

In contrast to this muscular uniformity, the distal tendon of the tibialis anterior (TAT) exhibits substantial morphological variability, especially regarding its insertion on the medial cuneiform and first metatarsal bones. Numerous studies have documented divergent insertion patterns, including bifid and trifid arrangements, as well as rare configurations with accessory slips or anomalous course (Musiał, 1963; Arthornthurasook and Gaew Im, 1990; Brenner, 2002; Olewnik et al., 2019; Karauda et al., 2021; Zielinska et al., 2021).

The aim of this review is to provide an integrative perspective on the TAT, encompassing developmental origin, morphological classifications in both adult and fetal populations, comparative and evolutionary anatomy, diagnostic imaging features, and clinical-surgical relevance.

## 2 Developmental anatomy

### 2.1 Embryological background

The TAM originates from the dorsal muscle mass of the lower limb bud, and its myogenic differentiation begins between the sixth and eighth week of gestation. Although TAM shares a common mesodermal progenitor with the extensor digitorum longus (EDL) and extensor hallucis longus (EHL) (Zielinska et al., 2021), initial muscle segregation becomes evident at the embryonic stage corresponding to a 14 mm crown–rump length, and a pattern consistent with the adult musculature is already recognizable in embryos measuring approximately 20 mm (Karauda et al., 2021; Zielinska et al., 2021).

These observations confirm the early ontogenetic establishment of the anterior crural compartment and suggest that variation of the TAT may stem from subtle modulations in the differentiation or apoptosis of tendon primordia within this period.

### 2.2 Fetal morphological classification

The developmental anatomy of the TAT was extensively studied by Karauda et al. (2021), who analyzed 100 lower limbs obtained from 50 spontaneously aborted fetuses between 18 and 38 weeks of gestation. Their analysis led to the establishment of a six-type classification (Types I–VI), based on distal insertion patterns.

• Type I: The tendon splits into two equal-sized bands inserting onto the medial cuneiform and base of the first metatarsal, respectively (19%).
• Type II: The tendon divides into two bands, with the wider band inserting onto the medial cuneiform and the narrower one onto the base of the first metatarsal (12%).
• Type III: Similar to Type II but with reversed width the narrower band attaches to the medial cuneiform and the broader to the first metatarsal base (5%).
• Type IV: Characterized by three distal insertions medial cuneiform, and both the base and shaft of the first metatarsal. This type was not observed in the fetal series, likely due to underdevelopment or sample limitations.
• Type V: A single band inserting solely onto the medial cuneiform; this was the most common type in fetuses, present in 60% of cases.
• Type VI: A unique variant identified only in fetuses, composed of three distinct bands one inserting onto the medial cuneiform, and two onto the base of the first metatarsal. This type does not correspond to any adult configuration and may represent a transitory embryological phenotype (4%).

This classification illustrates both the diversity and developmental specificity of TAT architecture during gestation. It provides a structural framework for understanding the ontogenetic origins of adult variation (Karauda et al., 2021; Zielinska et al., 2021).

A detailed summary of the six morphological types identified in the fetal population is presented in Table 1. Notably, Type V was the most prevalent, whereas Type IV was absent, and Type VI was exclusive to fetuses (Karauda et al., 2021).

**TABLE 1:** Morphological classification of the tibialis anterior tendon in human fetuses (n = 100).

Type	Description	Prevalence (%)
I	Two equal-sized bands inserting on medial cuneiform and base of first metatarsal	19
II	Wider band to medial cuneiform, narrower to base of first metatarsal	12
III	Narrower band to medial cuneiform, broader to base of first metatarsal	5
IV	Three bands: medial cuneiform, base and shaft of first metatarsal (not observed in fetuses)	0
V	Single band to medial cuneiform	60
VI	Three distinct bands: medial cuneiform and two to base of first metatarsal (unique to fetuses)	4

## 3 Adult morphological variability

### 3.1 Cadaveric classification

In a pivotal study conducted by Olewnik et al. (2019), one hundred formalin-fixed lower limbs (50 paired specimens) were examined to establish a detailed anatomical classification of the TAT in adults. The proposed classification consisted of five morphotypes (Types I–V), all confirmed through meticulous dissection and morphometric analysis.

• Type I: Two equal-sized bands inserting onto the medial cuneiform and base of the first metatarsal (31%).
• Type II: Wider band to the medial cuneiform and narrower to the first metatarsal (24%).
• Type III: Narrower band to the medial cuneiform and broader to the first metatarsal (11%).
• Type IV: Trifid tendon with three distinct insertions onto the medial cuneiform, and both the base and shaft of the first metatarsal. This type was rare, observed in only 2% of specimens.
• Type V: Single band inserting solely on the medial cuneiform bone, the most prevalent configuration (32%).

This classification not only integrated earlier typologies (Musiał, 1963; Arthornthurasook and Gaew Im, 1990; Brenner, 2002), but also refined the morphometric understanding of tendon architecture. Importantly, the study revealed significant differences in tendon width, thickness, and distance to insertion between types, which may have biomechanical and surgical relevance (Olewnik et al., 2019).

### 3.2 Ultrasonographic typing

A complementary ultrasonographic analysis of the TAT was performed by Olewnik et al. (2019) in a cohort of 50 healthy volunteers (23 women, 27 men; mean age 39 years). The sonographic study confirmed the presence of Types I–III and V previously identified in cadaveric specimens, but also revealed a novel variant—Type VI not observed in anatomical dissection.

This newly Type VI classification was characterized by two equal-sized tendinous bands, both inserting onto the medial cuneiform bone. It was detected in 12% of the subjects and is considered unique to imaging-based classification, likely due to the high resolution required to distinguish such subtle anatomical variants.

Interestingly, Type IV characterized by three distal insertions was not detected via ultrasound, possibly due to technical limitations in visualizing extremely thin slips or its genuinely low prevalence in the general population. Despite this, the sonographic classification demonstrates high diagnostic value and could be clinically relevant in preoperative planning and differential diagnosis of anterior ankle pathologies (Olewnik et al., 2019; Zielinska et al., 2021). Table 2 summarizes the frequency of TAT morphological types identified in fetal, cadaveric, and ultrasound-based studies, providing a developmental and methodological comparison.

**TABLE 2:** Comparative frequency of tibialis anterior tendon (TAT) types: Fetal, adult cadaveric, and ultrasound studies.

Type	Fetal study (%)	Adult cadaveric study (%)	Ultrasound study (%)
I	19	31	20
II	12	24	35
III	5	11	13
IV	0	2	0
V	60	32	20
VI	4	0	12

### 3.3 Morphometric and structural observations

Beyond qualitative classification, Olewnik et al. (2019) conducted a quantitative morphometric analysis of the TAT, revealing statistically significant differences in tendon length, width, and thickness among the identified types. These measurements were obtained using digital calipers during cadaveric dissection and included the width and thickness at the insertion site, as well as the distance from the tendon origin.

For example, the first band thickness ranged from 2.12 mm (in Type V) to 3.24 mm (in Type II), while the band width spanned from 5.10 mm to 10.65 mm, depending on the type. Additionally, the insertion distance varied significantly between morphotypes, suggesting distinct biomechanical implications in terms of lever arm function and tension dynamics across types (Olewnik et al., 2019).

These structural variations may influence force distribution across the medial foot and forefoot, with potential clinical relevance for tendon repair techniques, tension balancing, and the design of tendon grafts.

## 4 Topographic and layered anatomy

### 4.1 General compartmental organization

The TAT is the most anteromedial structure of the anterior crural compartment of the leg. It is flanked laterally by the EHL and EDL, with the fibularis tertius (FT) positioned even more laterally, when present. Medially, the tendon borders the subcutaneous surface of the tibia, often serving as a palpable landmark on physical examination or during surgical exposure (Moore et al., 2017).

### 4.2 Fascial relationships and retinacular architecture

As the TAT courses distally, it traverses under the superior extensor retinaculum, a broad transverse band extending from the tibia to the fibula, and then passes deep to the inferior extensor retinaculum, which forms a Y-shaped structure inserting into the calcaneus and medial malleolus. The tendon runs through a fibro-osseous tunnel formed by these structures, which prevents anterior displacement and bowstringing during dorsiflexion (Spence and Forro, 2020). Entrapment or synovial inflammation in this region may mimic early tendinopathy or compartmental syndrome.

### 4.3 Layered cross-sectional anatomy

At the level of the mid-leg, the TAT lies deep to the crural fascia and the subcutaneous fat, coursing in the first fascial layer. It is covered by skin, superficial fascia, and investing fascia before giving way to the underlying EDL and EHL. Distally, the tendon becomes more superficial as it approaches the medial border of the ankle, where it lies just deep to the retinacula. The second fascial layer consists of the neurovascular bundle, primarily the anterior tibial artery and the deep fibular nerve, which travel in a neurovascular plane between TAT and EDL (Arias and Marappa-Ganeshan, 2020). This layered relationship is crucial during flap elevation or compartment decompression.

### 4.4 Neurovascular proximity and surgical risk zones

The deep fibular nerve courses laterally to the TAT in the proximal and mid-leg, but due to rotational positioning of the leg and intercompartmental fascia, it becomes more anterior and crosses medially as it reaches the ankle joint. In surgical exposures, particularly during anterior fasciotomies or reconstructive tendon transfers, the zone between the inferior extensor retinaculum and the talonavicular joint presents a particular risk for iatrogenic neurovascular damage (Harkin et al., 2017; Willegger et al., 2017). Likewise, branches of the anterior tibial artery may form small nutrient vessels that penetrate toward the TAT’s synovial sheath.

### 4.5 Distal course and insertional topography

Distal to the retinacula, the TAT turns medially and inserts depending on the anatomical variant on the medial cuneiform, the base of the first metatarsal, or both. The angular transition, often at ∼90°, exposes the tendon to shear forces during inversion and dorsiflexion. In patients with bifid or trifid insertions (e.g., Types I–IV), the insertional footprint spans an oblique line from the medial cuneiform to the dorsal base of the first metatarsal (Olewnik et al., 2019; Zielinska et al., 2021). Awareness of this footprint is important during osteotomy, arthrodesis, or minimally invasive approaches to the medial column.

### 4.6 Surgical and radiological relevance

From a surgical standpoint, the TAT is a key anatomical landmark for anterior ankle arthrotomy, fasciotomy in acute compartment syndrome, and harvesting for tendon transfer procedures. It may also be accessed during anterior ankle impingement debridement, particularly when ossified retinacular structures or hypertrophied synovium obscure the tendon sheath. Given its superficial location and low variability in course (despite insertional variation), the TAT is considered both a reliable guide and a vulnerable structure in lower leg interventions. Radiologically, its intimate relation with the deep fibular nerve and anterior tibial artery must be recognized in cross-sectional imaging, especially in cases of trauma, pseudotumor evaluation, or suspected tendinopathy (Maharaj et al., 2019; Olewnik et al., 2019; Zielinska et al., 2021).

## 5 Comparative anatomy and evolutionary trajectories

### 5.1 Early vertebrates and basal tetrapods

In actinopterygians, paired-fin musculature is organized into primitive dorsal/ventral masses without a discrete TAT analogue (Diogo, 2010; Diogo and Abdala, 2010). Lobe-finned sarcopterygians (e.g., *Latimeria*) show more internal division of appendicular muscles, but still lack a tendon with the distal, medial foot insertion and dorsiflexion-specific leverage characteristic of amniote TAT (Diogo et al., 2009; Diogo and Wood, 2012). Embryology supports a tetrapod origin: human TAT derives from the dorsal muscle mass of the lower limb bud an arrangement not differentiated in fish (Bardeen and Lewis, 1901; Bardeen, 1906).

Extant amphibians (e.g., *Xenopus*) possess generalized dorsal crural extensors/dorsiflexors with short aponeurotic insertions and no distinct bifurcation to the medial cuneiform/first metatarsal (MT1); thus any TAT relation is positional rather than morphological (Diogo, 2007; Abdala and Diogo, 2010). Overall, a discrete, distally inserting TAT is best interpreted as a post-amphibian innovation linked to controlled ankle dorsiflexion and medial foot stabilization during early terrestrial gait (Diogo and Abdala, 2010; Diogo et al., 2019).

### 5.2 Reptiles

#### 5.2.1 Squamata (lizards, snakes) — simple dorsiflexors, Type V analogue

In limbed squamates (e.g., *Varanus*, *Anolis*), a prominent tibialis externus contributes to ankle dorsiflexion and typically inserts as a single, undivided band on the medial tarsus/proximal metatarsals an analogue of human Type V; trifid/bifid terminals are not reported (Heilmann, 1926; Diogo and Wood, 2012; Diogo & Abdala, 2010a). Hindlimb-regressed snakes lack a useful homologue. Functionally, this single-unit pattern fits sprawled gait mechanics, where precise medial stabilization is less critical. See Table 3 for a reptilian overview.

**TABLE 3:** Comparative overview of reptilian dorsiflexors and human tibialis anterior tendon analogs.

Group	Representative muscles	Tendon insertion	Distal branching	Orientation	Analogous human type
Squamata	m. tibialis externus	Single-band, medial tarsus	Absent	Straight, craniomedial	Type V
Testudines	m. tibialis externus, m. ext. digitorum longus	Broad aponeurotic, dorsomedial tarsus	Absent	Medialised, rotated	Modified Type V

#### 5.2.2 Testudines (turtles) — medialised dorsiflexors, functionally repositioned

Turtle hindlimb dorsiflexors (tibialis externus and parts of EDL) are medially shifted with broad, aponeurotic insertions; tendon duplication is unreported (Diogo, 2007; Diogo and Abdala, 2007, 2010). Rotational reorientation of limb axes within a rigid trunk yields topographic/biomechanical repositioning rather than added distal complexity—again a modified Type V analogue. See Table 3.

### 5.3 Birds—comparative insights

Across birds, the tibialis cranialis (homologue of human TAM) is highly plastic. Galliformes (e.g., *Gallus*) commonly show distal bifurcation with slips to the medial tarsometatarsus and proximal MT1—Type I/II analogues supporting fine medial control during terrestrial bipedalism/perching (Bardeen and Lewis, 1901; Diogo and Abdala, 2010; Diogo et al., 2019). Accipitriformes (e.g., *Aquila*, *Buteo*) exhibit a hypertrophied, reinforced tendon that functionally differentiates medial/lateral components for stabilization and grasp—akin to a reinforced Type II solution (Diogo et al., 2009; Diogo and Wood, 2012; Apaydin et al., 2008). In Sphenisciformes (penguins), the tendon is short, embedded, and proximally/laterally redirected, acting chiefly as a stabilizer—Type V-like but functionally decoupled from active dorsiflexion (Diogo et al., 2009; Diogo and Abdala, 2010; Diogo et al., 2019). See Table 4 for avian morphotypes and human analogues.

**TABLE 4:** Comparative avian tibialis cranialis tendon morphotypes and human analogs.

Avian order	Representative species	Tendon structure	Insertion pattern	Functional role	Analogous human type
Galliformes	Gallus gallus	Bifid	Medial tarsometatarsus and metatarsal I	Ground locomotion, medial control	Type I/II
Accipitriformes	*Aquila chrysaetos*/Buteo spp.	Hypertrophied (sometimes functionally bifid)	Reinforced insertion on tarsometatarsus and hallux base	Power grasping, talon stabilization	Reinforced Type II
Sphenisciformes	Aptenodytes forsteri/Pygoscelis papua	Reduced, short and embedded	Reoriented, proximal or lateral insertion on tibiotarsus	Passive postural support on land	Type V (functionally regressed)

### 5.4 Mammals

#### 5.4.1 Rodentia—short, simple TAT (Type V)

Plantigrade rodents typically retain a single, robust band inserting on the medial cuneiform or MT1 base, without distal branching—morphologically/functionally analogous to Type V (Heilmann, 1926; Diogo and Abdala, 2010; Diogo et al., 2019). Limited distal subdivision and a short tendinous segment fit rapid, unspecialized paw placement.

#### 5.4.2 Carnivora—elongated, slender TAT with stable insertion (± accessory slips)

Digitigrade cats/dogs show a long, slender TAT inserting on the medial tarsus/MT1; occasional fascial expansions occur, but true bifurcation (Type I/II) is uncommon (Heilmann, 1926; Diogo and Abdala, 2010). The tendon acts as a precise vector stabilizer without the complexity of multiple slips (Apaydin et al., 2008; Diogo and Wood, 2012).

#### 5.4.3 Artiodactyla—reduced or repositioned TAT with proximal insertion

Ungulates show small or proximally inserting tendons (upper tarsus/medial malleolus), often vestigial in function, reflecting unguligrade mechanics dominated by elastic/digital flexor systems; a truncated Type V analogue best fits this organization (Heilmann, 1926; Diogo and Abdala, 2010; Diogo et al., 2019).

#### 5.4.4 Primates—high variability; humans robust and often bifid (types I–III)

Primates range from single-band Type V/II-like insertions in arboreal taxa (*Macaca*, *Cebus*) to bifid/trifid terminals in great apes and especially humans, inserting on the medial cuneiform and MT1 base/shaft—Types I–IV (Olewnik et al., 2019; Diogo et al., 2009; Diogo and Wood, 2012). Human bifid forms support dorsiflexion plus fine medial stabilization during bipedal stance (Jungers et al., 1993; Jana and Roy, 2011).

#### 5.4.5 Chiroptera—reduced/reoriented TAT

Bats often show a highly reduced, short, non-bifid tendon with proximal/medial tarsal insertion or fascial fusions, reflecting suspensory posture and passive locking; active dorsiflexion is minimized—regressed Type V analogue. Details in Supplementary Text S1 (Diogo et al., 2009; Diogo and Abdala, 2010; Diogo and Wood, 2012; Heilmann, 1926).

#### 5.4.6 Cetacea—complete regression of TAT

Modern whales/dolphins lack external hindlimbs; thus TAT is absent (terminal anatomical loss). Fossil forms retain vestiges, but no functional tendon persists in extant taxa. Details in Supplementary Text S2 (Diogo, 2007; Diogo and Abdala, 2010; Diogo and Wood, 2012).

See Table 5 for the full mammalian overview.

**TABLE 5:** Full comparative overview of tibialis anterior tendon morphology in mammals.

Mammalian order	Representative species	TAT structure	Insertion pattern	Functional role	Analogous human type
Rodentia	*Rattus norvegicus*	Short, simple, undivided	Medial cuneiform or 1st metatarsal base	Basic dorsiflexion, limb repositioning	Type V
Carnivora	*Felis catus*/*Canis lupus*	Elongated, slender, ± accessory slips	Medial tarsus/1st metatarsal	Precise dorsiflexion, digitigrade stance	Type V (elongated)
Artiodactyla	*Ovis aries*/*Cervus elaphus*	Reduced, proximally inserting	Upper tarsus/medial malleolus	Minimal dorsiflexion, passive stabilization	Truncated Type V
Primates	*Homo sapiens*/Macaca spp.	Variable; bifid/trifid in humans	Medial cuneiform +1st metatarsal (base/shaft)	Bipedal stability, medial foot control	Types I–IV
Chiroptera	Pteropus spp./Myotis spp.	Reduced or reoriented; passive	Short/fused insertion near tarsus	Suspensory support, passive locking	Regressed Type V
Cetacea	*Delphinus delphis*/Balaenoptera musculus	Absent (complete regression)	No insertion; limb structure regressed	No functional role; limb vestigial	None (absent)

### 5.5 Evolutionary and evo-devo summary

Across vertebrates, a single-band, medially inserting tendon represents the conservative template (Type V analogue) retained in squamates, many mammals (rodents, carnivores), and simplified avian or aquatic specializations. Progressive distal complexity with bifid/trifid terminals emerges in lineages requiring refined medial control or manipulative precision (terrestrial bipedal birds, raptorial birds functionally; primates/humans anatomically), consistent with increasing demands on arch support, dorsiflexion efficiency, and forefoot stability (Diogo et al., 2009; Diogo and Abdala, 2010; Diogo and Wood, 2012).

Embryologically, the TAT originates from the dorsal muscle mass, with late-fetal fascicular divergence underpinning distal patterning (Bardeen and Lewis, 1901; Bardeen, 1906; Diogo et al., 2019). This modular architecture (HOX/Tbx/Scx-mediated) explains how small shifts in developmental domains yield stable single-band vs. split-tendon outcomes with minimal change to overall topology (Diogo and Abdala, 2010).

In humans, Types I–II (bifid) plausibly reflect bipedal gait stabilization by distributing forces across the medial cuneiform and MT1, improving energy transfer and mitigating local stress (Olewnik et al., 2019; Zielinska et al., 2021; Jungers et al., 1993). Type VI (double band to the medial cuneiform) appears benign and relatively common in imaging cohorts, likely arising from incomplete fascicular regression or neutral duplication; whether it enhances medial support is a testable hypothesis for future kinematic work (Olewnik et al., 2019; Karauda et al., 2021).

Taken together, TAT variability is a morpho-functional signature of evolutionary pressures: distal tendon complexity increases where fine control is advantageous, while regression accompanies diminished dorsiflexion roles (e.g., ungulates, bats, cetaceans). Table 6 summarizes presence, insertion patterns, and functional roles across representative groups.

**TABLE 6:** Morphological types of tibialis anterior tendon (TAT) across representative vertebrates.

Taxonomic group	TAT presence	Insertion pattern	Functional role
Fish (e.g., Latimeria)	Absent	Absent	None
Amphibia (*Xenopus laevis*)	Present (primitive)	Short fascicle; proximal tarsus	Basic dorsiflexion
Reptilia – Squamata	Present (simple)	Single band; medial tarsus	Simple dorsiflexion
Reptilia – Testudines	Present (medialised)	Medial tarsus; repositioned	Modified support
Aves – Galliformes	Present (bifid)	Dual slips; Type I/II analog	Grasping, perching
Aves – Accipitriformes	Present (hypertrophied)	Robust slip; grasping digit	Forceful grasping
Mammalia – Rodentia	Present (short)	Single band; Type V	Limb repositioning
Mammalia – Carnivora	Present (elongated)	Elongated band; modified Type V	Precise dorsiflexion
Mammalia – Artiodactyla	Present (reduced)	Truncated band; proximal insertion	Passive stabilization
Mammalia – Chiroptera	Present (reoriented)	Short/fused; regressed Type V	Suspensory locking
Mammalia – Cetacea	Absent	No insertion (hindlimb lost)	No role (regressed limb)
Mammalia – Primates	Present (variable)	Bifid/trifid; Types I–IV and VI	Bipedal control, balance

## 6 Functional biomechanics

### 6.1 TAT as a dynamic stabilizer of the medial longitudinal arch

The TAT serves as a dynamic stabilizer of the medial longitudinal arch (MLA), a function that is particularly relevant during phases of gait requiring load absorption and medial support. Its insertion onto the medial cuneiform and first metatarsal provides a biomechanical anchor capable of counteracting ground reaction forces that tend to depress the arch during the loading response and midstance phases (Day et al., 2013; Maharaj et al., 2019).

TAT activity contributes to medial foot elevation and helps maintain midfoot alignment against pronatory forces. In patients with tibialis posterior insufficiency, the TAT is often secondarily overloaded, highlighting its compensatory capacity. Conversely, in conditions such as pes planovalgus or Charcot-Marie-Tooth disease, TAT dysfunction can contribute to collapse of the MLA and loss of medial support, with downstream consequences for proximal joints.

Notably, the variation in TAT insertion pattern may modulate this stabilizing function: bifid or trifid insertions may offer broader force distribution, enhancing load dissipation across the tarsometatarsal region (Olewnik et al., 2019; Zielinska et al., 2021).

### 6.2 Influence on dorsiflexion vector and gait mechanics

The TAT is the primary dorsiflexor of the ankle and foot, responsible for lifting the medial forefoot during the swing phase and controlling foot descent during heel strike. Beyond this sagittal function, however, the tendon also modulates frontal and transverse plane motions via its medial insertion orientation, influencing inversion and slight internal rotation of the forefoot (Apaydin et al., 2008; Maharaj et al., 2019).

During normal gait, TAT contraction peaks at terminal swing and early stance, providing a preparatory tension that enhances foot placement accuracy. Any asymmetry or morphological variation in its insertion (e.g., dominance of medial vs. lateral slip) can alter the direction and amplitude of the force vector, which may in turn predispose individuals to functional hallux limitus, overpronation syndromes, or stress-related medial foot pain.

Furthermore, high-resolution sonographic studies show that Type VI configurations, with dual bands inserting onto the medial cuneiform, may alter the effective lever arm of dorsiflexion and shift the line of pull closer to the subtalar axis. This could hypothetically result in decreased torque generation or altered proprioceptive feedback in dynamic loading scenarios (Bianchi et al., 2007; Olewnik et al., 2019).

### 6.3 Functional advantage of bifid insertions: a biomechanical hypothesis

The bifid and trifid insertion patterns of the TAT especially Types I through III may provide a functional advantage in mediolateral foot control, a concept especially relevant in humans due to the demands of bipedal locomotion (Jungers et al., 1993).

In contrast to simple insertions (Type V), bifid morphotypes allow the distribution of contractile force over a larger area, engaging both the medial cuneiform and various regions of the first metatarsal shaft and base. This may enable micro-adjustments of inversion/eversion loading, enhancing stance phase stability during single-limb support and improving the propulsive toe-off vector.

The presence of these types in both fetal and adult specimens suggests a developmentally programmed advantage, rather than a postnatal adaptation (Karauda et al., 2021). Moreover, their asymmetric distribution as documented by Olewnik et al. (Olewnik et al., 2019) may indicate laterality-driven specialization (e.g., dominant foot stabilization).

In evolutionary terms, such bifid types could have emerged as a functional specialization linked to upright posture, where precise control of foot orientation is crucial for gait efficiency and fall prevention (Diogo and Wood, 2012; Zielinska et al., 2021).

### 6.4 Type-specific functional implications (analytical synthesis)

Rationale. Distal insertion patterns of the tibialis anterior tendon (TAT) modulate (i) the net line of pull relative to the talocrural/subtalar axes, (ii) load distribution across the medial cuneiform (MC)–first metatarsal (MT1) complex, and (iii) first-ray kinematics during stance and push-off (Hicks, 1954; Inman, 1976; Brand and Hollister, 1999; Nordin and Frankel, 2001; Perry and Burnfield, 2010).

#### 6.4.1 Type I (two balanced slips to MC and MT1 base)

Wider, two-point footprint disperses tensile loads across MC–MT1 and provides strong pronation control under load; the MT1 slip adds a distal–dorsal component improving first-ray dorsiflexion leverage, while the MC slip preserves medial bracing net effect: stable arch support with reduced local stress concentration (Hicks, 1954; Inman, 1976; Brand and Hollister, 1999).

#### 6.4.2 Type II (MC-dominant + thin MT1 slip)

Resultant vector shifts more medially and the inversion moment increases; useful where over-pronation dominates, but in feet with a supination bias may accentuate medial-column rigidity unless counterbalanced (Nordin and Frankel, 2001; Perry and Burnfield, 2010).

#### 6.4.3 Type III (MT1-dominant + thin MC slip)

A more distal/dorsal line of pull augments first-ray elevation and may facilitate toe clearance, but can increase lateralising forces at the hallux in hallux-valgus phenotypes when the MT1 slip is long/stiff; consider vector re-direction if planning a tendon transfer (Perry and Lafortune, 1995; Nordin and Frankel, 2001; Perry and Burnfield, 2010).

#### 6.4.4 Type IV (trifid, rare)

The broadest distal dispersion yields maximal load spreading and redundancy. Functional advantage is nuanced (stability), but surgical exploitation is non-trivial: higher risk of incomplete harvest without pre-operative mapping (Bianchi and Martinoli, 2007; Olewnik et al., 2019).

#### 6.4.5 Type V (single monotendinous to MC)

Predictable line of pull and robust medial bracing; the smaller footprint implies higher local stress density at peak loads, yet this configuration is generally the most straightforward for harvest/transfer with fewer conflict zones (Inman, 1976; Olewnik et al., 2019).

#### 6.4.6 Type VI (double bands to MC)

Duplicated, closely spaced medial vectors act like a buttress to the medial column, with a potentially shorter effective dorsiflexion moment arm vs. Type I/III but enhanced medial stiffening. Imaging cohorts indicate a benign, relatively frequent variant without adverse clinical correlation (Olewnik et al., 2019; Karauda et al., 2021; Zielinska et al., 2021).

#### 6.4.7 Implications for assessment

On US/MRI, reports should state Type (I–VI), side, dominant insertion(s), and expected vector biases (medial bracing vs. distal dorsiflexion leverage). For surgical planning (transfer, HV correction), map usable length and slip calibre; for Types I–IV, anticipate fascicular heterogeneity during harvest (Bianchi and Martinoli, 2007; Olewnik et al., 2019).

## 7 Imaging features and diagnostic pitfalls

### 7.1 MRI and ultrasound can distinguish tibialis anterior tendon morphotypes

Modern imaging techniques, particularly magnetic resonance imaging (MRI) and diagnostic musculoskeletal ultrasound (US), are capable of differentiating between various morphotypes of the TAT, especially Types I through VI. High-resolution US has been validated for identifying insertional variants, with Type VI defined by a double fascicular band inserting solely into the medial cuneiform being visualized in approximately 6% of healthy individuals (Olewnik et al., 2019).

On MRI, bifid or trifid tendons appear as parallel low-signal structures on axial and coronal T1 and T2 sequences, with clearly delineated margins and no signs of peritendinous edema. These features enable the non-invasive classification of TAT morphology *in vivo*, providing a direct radiological correlate to anatomical classifications (Lee et al., 2006; Zielinska et al., 2021).

### 7.2 Diagnostic pitfalls: misinterpretation of bifidTAT as tendinopathy or tear

A frequent pitfall in TAT imaging lies in misinterpreting normal anatomical variants as pathology. For instance, bifid tendons (Types I–III) may mimic partial-thickness tears or degenerative tendinopathy, particularly on axial US, where they may appear as hypoechoic clefts between tendon slips (Lee et al., 2006; Bianchi et al., 2007).

A 2022 prospective US study demonstrated that over 80% of “suspected partial tears” in the region 2–3 cm proximal to the insertion were normal bifid variants when confirmed by cadaveric comparison (Pośnik et al., 2023). Similarly, in MRI, a split TAT with clean fascicular continuity and no adjacent fluid should not be interpreted as pathology (Zielinska et al., 2021).

### 7.3 Recognition of Type VI in US as non-pathological

Among the most critical insights is the need to accurately identify Type VI—a dual-banded TAT insertion limited to the medial cuneiform as a benign anatomical variant. Studies by Olewnik et al. (2019) and Karauda et al. (2021) have shown that Type VI is found in up to 4%–6% of the general population, often bilaterally, and bears no clinical correlation with pain or dysfunction.

Key ultrasonographic features of Type VI include two symmetrical hyperechoic bands of equal thickness, preserved fibrillar alignment, and absence of adjacent inflammatory changes. Incorrect labeling of this configuration as a partial rupture or longitudinal tear may lead to unnecessary interventions such as immobilization, corticosteroid injection, or even surgical exploration (Zielinska et al., 2021).

The principal imaging features and diagnostic pitfalls associated with each TAT type are summarized in Table 7.

**TABLE 7:** Imaging features and diagnostic confounders by TAT type.

TAT type	Imaging characteristics (US/MRI)	Potential diagnostic pitfall
I–III	2–3 well-defined slips with preserved continuity	Mistaken for longitudinal split or partial tear
IV	Absent in most adults, not commonly visualized	May be wrongly interpreted as hypoplasia or pathology
V	Single homogeneous band	May appear thickened or nodular in a degenerative context
VI	Two equal fascicles, insertion on the medial cuneiform	Misdiagnosed as tear, synovial cyst, or tendinosis

Clinical implications include the need for radiologists and musculoskeletal sonographers to recognize the anatomical variability of the TAT to avoid overdiagnosis of benign patterns. Accurate identification of Type VI as a non-pathological variant is particularly important in asymptomatic individuals. Additionally, dynamic ultrasonography during active dorsiflexion and correlation with cross-sectional imaging should always be interpreted in the context of the clinical presentation to reliably distinguish anatomical variants from true pathology.

## 8 Clinical and surgical relevance

### 8.1 Tendon harvest in foot drop: Types V and VI are the safest options

In the surgical management of foot drop, particularly in cases requiring tendon transfer procedures, the TAT is frequently considered for harvest or rerouting (Sammarco et al., 2009; Harkin et al., 2017). However, not all morphotypes are equally suited for safe and effective transfer.

Type V, characterized by a single robust insertion to the medial cuneiform and/or base of the first metatarsal, provides a consistent and easily dissectible structure. Likewise, Type VI, with two symmetrical bands inserting solely into the medial cuneiform, offers predictable anatomy with minimal neurovascular conflict zones (Olewnik et al., 2019). These types ensure sufficient tendon length and strength for rerouting and anchoring in new trajectories, such as to the lateral cuneiform or cuboid in post-polio or peroneal nerve palsy patients.

Conversely, Types I–III with bifid or trifid insertions can complicate harvest due to fascicular divergence, increased risk of incomplete transfer, or iatrogenic partial injury to one of the slips.

### 8.2 Importance of insertion mapping before reconstruction or tendon transfer

Detailed knowledge of the insertion pattern of the TAT is essential prior to planning surgical tendon transfers, reconstructions, or correction of hallux valgus (Brenner, 2002; Willegger et al., 2017). Given the wide morphological variability, preoperative mapping using high-resolution US or MRI should be considered the standard of care.

Insertional diversity not only affects the length and thickness of available tendon tissue but also influences its pull vector and mechanical efficiency. For example, failure to recognize a bifid Type II insertion may result in an underpowered correction due to inadequate force transfer through an incomplete harvest.

Additionally, hallux valgus surgery may benefit from insertion-type awareness: studies show that the directional pull of Type I or II configurations may accentuate deformity postoperatively if not redirected properly (Brenner, 2002; Zielinska et al., 2021).

### 8.3 Diagnostic ultrasound in postoperative monitoring and rehabilitation stratification

US is a powerful tool not only in preoperative planning, but also in postoperative monitoring of TAT reconstructions or rerouted transfers. Dynamic US can visualize tendon continuity, adhesion formation, rerouting integrity, and neovascularization, providing early insight into recovery or complication onset (Pośnik et al., 2023).

Moreover, in postoperative rehabilitation, ultrasound-guided assessments can stratify patients into risk-based recovery protocols, especially when dealing with asymmetrical insertion types, where one slip may exhibit delayed remodeling or fibrosis.

Routine US at 4–6 weeks and again at 12 weeks post-surgery can aid in optimizing physical therapy regimens, detecting early complications, and guiding clinical decisions regarding return to activity.

### 8.4 Clinical implementation

#### 8.4.1 Imaging acquisition

Ultrasound (US). High-frequency MSK US (12–18 MHz) in long- and short-axis planes along the TAT, from the retinacular region to the distal insertions at the medial MC and first metatarsal (MT1), enables identification of distal slips and their continuity. Dynamic manoeuvres (active/passive dorsiflexion and inversion/eversion) increase sensitivity for split tendons and reduce anisotropy-related artefacts (Bianchi and Martinoli, 2007; Pośnik et al., 2023).

MRI. Proton-density fat-suppressed (PD-FS) sequences aligned with the tendon’s course (long-axis), complemented by axial images under the extensor retinaculum and coronal images through MC/MT1, allow confirmation of fascicular integrity and mapping of distal insertion patterns (Lee et al., 2006).

#### 8.4.2 Reporting standards

To support reproducible research and clinical decision-making, radiology reports should include a minimum dataset: Type (I–VI), side, dominant insertion(s) (MC; MT1 base/shaft), fascicular integrity (intact/tear), peritendinous tissue reaction (fluid/edema), and a brief clinical note indicating potential implications for surgery or rehabilitation (Olewnik et al., 2019; Zielinska et al., 2021). For uncommon morphotypes (e.g., Type IV), the report should state “variant—not a tear” when continuity is preserved and no inflammatory signs are present (Lee et al., 2006; Bianchi and Martinoli, 2007).

#### 8.4.3 Surgical planning and intervention

Pre-operative mapping of the TAT (type and usable length) is recommended before tendon transfer, reconstruction, or first-ray procedures. Neurovascular relationships in the retinacular region (deep fibular nerve; anterior tibial artery) should be documented to reduce iatrogenic risk (Arias and Marappa-Ganeshan, 2020; Spence and Forro, 2020). Knowledge of the distal footprint informs screw/osteotomy placement around MC/MT1 (Willegger et al., 2017). Type-aware considerations derived from the analytical synthesis in Section 6.4 include: load sharing across MC–MT1 in Types I–II; more distal/dorsal leverage and possible hallux valgus interactions in Type III; harvest complexity in Type IV; and predictability for transfer in Types V–VI. Management of ruptures and chronic tendinopathy should integrate morphology with tissue quality and continuity when selecting repair, augmentation, or transfer techniques (Sammarco, 2009; Harkin et al., 2017).

#### 8.4.4 Quality assurance and reader reliability

Given historical variability in nomenclature and recognition of rare morphotypes, centres implementing the classification should report inter- and intra-observer reliability (e.g., ICC) for type assignment and distal slip identification, and utilise a shared image atlas with borderline examples to harmonise training (Zielinska et al., 2021).

#### 8.4.5 Common interpretative pitfalls

Bifid/trifid variants may mimic partial tears when anisotropy or retinacular coverage obscures fascicular continuity; dynamic US and plane-aligned MRI mitigate this risk (Lee et al., 2006; Bianchi and Martinoli, 2007; Pośnik et al., 2023). Under-sampling of the distal MT1 shaft region can lead to missed Type III slips; targeted distal sweeps are advised. Over-calling rare types without dynamic corroboration should be avoided.

#### 8.4.6 Implementation summary (for rapid adoption)

Reports using the proposed classification should include a one-line summary: “TAT Type (I–VI), side, dominant insertions (MC/MT1 base/shaft), integrity, peritendinous findings, and clinical remark.” In operative planning, teams should confirm type and length on imaging, protect the deep fibular nerve and anterior tibial artery during exposure, and reassess the post-transfer line of pull to avoid unintended inversion bias (Arias and Marappa-Ganeshan, 2020; Spence and Forro, 2020).

## 9 Proposed unified classification framework

Despite the anatomical constancy of the TAM belly, its tendon exhibits remarkable variability in distal insertion. Historically, disparate classification systems have hampered unified interpretation across anatomical, radiological, and surgical domains. In this review, we advocate for the implementation of a standardized six-type classification originally proposed by Olewnik et al. (2019) which integrates findings from fetal dissections, cadaveric studies, and high-resolution ultrasonography.

### 9.1 Morphotypes I–VI: integrated and clinically applicable

The six-type model, grounded in cadaveric evidence and validated by imaging and developmental data, captures the full morphological spectrum of TAT insertion. Types I through IV include bifid and trifid configurations with variable dominance between medial cuneiform and the base of the first metatarsal, while Types V and VI are characterized by simplified, medial insertions.

Table 2 presents a comprehensive summary of all morphotypes, including their anatomical characteristics, insertion topography, population frequency, and surgical implications.

Importantly, Type V (single robust slip to the medial cuneiform) and Type VI (dual symmetrical slips, medial only) represent the most reliable morphotypes for tendon harvest, while Types I–IV, particularly trifid Type IV, may pose technical challenges or require more cautious surgical planning.

### 9.2 Proposal for standard adoption

Given its embryological foundation, descriptive clarity, and cross-platform reproducibility (via US and MRI), the classification proposed by Olewnik et al (Olewnik et al., 2019) provides a robust and comprehensive framework for understanding TAT variability. Its clinical applicability has been demonstrated in tendon transfer planning, rupture repair, and radiological differentiation of anatomic variants from pathology.

We propose the universal adoption of this classification system in:

• Surgical protocols (e.g., tendon rerouting, foot drop correction),
• Educational settings (anatomy courses, surgical anatomy texts),
• Radiological diagnostics (differentiation of Type VI vs. partial tear).

Recommended adoption points (concise):

• Radiology reports: always include Type I–VI, side, dominant insertions (MC/MT1 base/shaft), and surgical implications (see Section 8.4.2).
• Pre-operative mapping: mandatory before TAT transfers and hallux valgus (HV) procedures; confirm type and usable length, and map DFN/ATA relationships (see Section 8.4.3).
• Education: in atlases/textbooks, include a schematic of distal footprints and common interpretive pitfalls (tear vs. bifid/trifid) with imaging examples (see Sections 7 and Sections 8.4.5).

## 10 Future directions and study limitations

### 10.1 Future directions

To close current evidence gaps and standardise methods across cohorts and modalities, we propose:

• Histology–imaging correlation. Correlate enthesis microstructure and collagen architecture across Types I–VI with US/MRI to validate footprint differences and fascicular continuity (Olewnik et al., 2019; Zielinska et al., 2021).
• 3D morphometrics and modelling. Use 3D segmentation (MRI/US) and photogrammetry to quantify footprint area on MC/MT1, tendon slip angles, and relationships to the extensor retinaculum; apply FEA/*in silico* simulations to estimate stress density and first-ray lever arms by type (Inman, 1976; Nordin and Frankel, 2001; Lee et al., 2006; Bianchi and Martinoli, 2007; Perry and Burnfield, 2010).
• Standardised imaging and reporting. Prospective, preregistered, multi-centre series with unified US (12–18 MHz; long/short axis; dynamic manoeuvres) and MRI (PD-FS/T2 in planes aligned to TAT), and a mandatory report line: Type I–VI, side, dominant insertions, and clinical implications [Section 8.4; Lee et al. (2006), Bianchi and Martinoli (2007), Pośnik et al. (2023)].
• Reader reliability. Require inter-/intra-observer ICC for type assignment and usable tendon length; develop a reference atlas with borderline examples (Karauda et al., 2021; Zielinska et al., 2021).
• Developmental trajectory. Link fetal, paediatric, and adult series to test the hypothesis of late-fetal fascicular divergence and postnatal stabilisation of type (Olewnik et al., 2019; Karauda et al., 2021).
• Functional correlation. Couple type with first-ray kinematics, pronation/supination, hallux valgus and forefoot pain using gait analysis and tendon energy-absorption metrics (Perry and Burnfield, 2010; Day et al., 2013; Maharaj et al., 2019).
• Interventional outcomes. In surgical cohorts (and RCTs where feasible), stratify outcomes and complications by anatomical type for tendon transfers and reconstructions (Sammarco, 2009; Harkin et al., 2017).

*Cross-references:* see Section 6.4 for type-specific biomechanical implications, and Section 8.4 for the reporting template and operative checklist.

### 10.2 Study limitations

This unified framework synthesises fetal, cadaveric and imaging datasets; several limitations remain:

1. Cohort and design heterogeneity. Differences in sampling (fetal vs. adult), setting (dissection vs. clinical imaging), and inclusion criteria limit comparability and may distort apparent type frequencies (Olewnik et al., 2019; Karauda et al., 2021; Zielinska et al., 2021).
2. Fixation/post-mortem artefacts. Formalin shrinkage and dehydration reduce linear measures and thickness, especially in small slips; morphometry is best interpreted within modality rather than across modalities.
3. Imaging–dissection discrepancies. US/MRI artefacts (anisotropy, retinacular cover, plane selection) can obscure thin slips or mimic splits/tears; conversely, dissection may over-resolve fascicles below clinical imaging resolution (Lee et al., 2006; Bianchi and Martinoli, 2007; Olewnik et al., 2019; Pośnik et al., 2023).
4. Reader reliability under-reported. Few studies provide inter-/intra-observer ICC for type assignment, limiting reproducibility and meta-analytic pooling (Zielinska et al., 2021).
5. Fetal-to-adult extrapolation. Developmental series inform ontogeny but cannot be directly extrapolated to adult prevalence/biomechanics without longitudinal or age-matched data (Olewnik et al., 2019).
6. Laterality and demographics. Effects of side dominance, sex, and ethnicity are underpowered or unreported, precluding robust stratified estimates (Brenner, 2002; Willegger et al., 2017).
7. Clinical selection bias. Surgical/symptomatic series may over-represent pathology-adjacent anatomy; imaging cohorts may reflect referral patterns (Sammarco, 2009; Harkin et al., 2017).
8. Terminology inconsistency. Historical synonyms (bifid/trifid) and variable labels complicate retrieval and synthesis; this review harmonised terminology (Harkin et al., 2017; Zielinska et al., 2021).
9. Limited functional linkage. Few datasets relate types to gait-level outcomes or first-ray mechanics; existing evidence supports a meaningful tendinous contribution but is not yet type-specific (Day et al., 2013; Maharaj et al., 2019).
10. Protocol heterogeneity. Variation in probe frequency, foot position, and MR planes hampers comparability and detection of rare types; we propose minimum parameters and a reporting line in Section 8.4 (Lee et al., 2006; Bianchi and Martinoli, 2007; Pośnik et al., 2023).
11. External validation outstanding. The classification and its clinical claims require prospective, preregistered validation with standardised protocols and blinded multi-reader assessment.

Mitigation within this review. We triangulated fetal/cadaveric/imaging evidence, emphasised within-modality comparisons for morphometrics, harmonised nomenclature, and provided a practical reporting template and operative checklist (Sections 7–8) to facilitate standardised future reporting (Olewnik et al., 2019; Karauda et al., 2021; Zielinska et al., 2021; Pośnik et al., 2023; Olewnik et al., 2019; Karauda et al., 2021; Zielińska et al., 2021; Pośnik et al., 2023).

## 11 Conclusion

The TAT demonstrates consistent yet structured morphological variability, rooted in embryological development and refined by functional demands. By integrating data from fetal dissections, adult cadavers, and high-resolution imaging, we propose a six-type classification system that is anatomically grounded and clinically useful.

This framework, originally described by Olewnik et al., offers practical value in surgical planning, radiological interpretation, and anatomical education. Its adoption may reduce diagnostic errors, guide tendon transfer strategies, and promote a deeper understanding of functional anatomy of the anterior compartment.
